# Power saw noise levels during steel stud cutting tasks on commercial construction sites: a tool characterization from a worker exposure standpoint

**DOI:** 10.1093/annweh/wxae054

**Published:** 2024-06-24

**Authors:** David Schutt, Tiffany Lipsey, Mike Van Dyke, William J Brazile

**Affiliations:** Department of Environmental and Radiological Health Sciences, Colorado State University, 1681 Campus Delivery, Fort Collins, CO, 80523-1681, United States; Department of Health and Exercise Science, Colorado State University, 1582 Campus Delivery, Fort Collins, CO, 80523-1582, United States; Department of Environmental and Occupational Health, Colorado School of Public Health, CU Anschutz Medical Campus, Aurora, CO, 80045, United States; Department of Environmental and Radiological Health Sciences, Colorado State University, 1681 Campus Delivery, Fort Collins, CO, 80523-1681, United States

**Keywords:** construction hazards, noise exposure, power tools, steel studs

## Abstract

Construction framers who cut and install steel studs as part of their daily tasks are exposed to hazardous noise levels during their work shift in large part due to the power saws they use to cut steel studs. This investigation characterized the sound pressure levels of power saws used to cut steel studs on active construction sites. Further, the length of time it took to cut various studs on a construction site was investigated to understand worker exposure times to saw noise. In general, power saws used on the study sites to cut steel studs had a mean A-weighted equivalent continuous sound pressure level (L_Aeq_) of 107.2 dB and a C-weighted peak sound pressure level (L_Cpeak_) of 120.1 dB. Three of the saws—the chopsaw, the cut-off saw, and the grinder—had similar noise levels, whereas the cordless circular saw had higher noise levels. It took an average of 13.2 s to cut each stud, and workers in the study used power saws to cut steel studs for an average of 371.5 s per day. This average exposure time at the average recorded sound pressure levels (SPLs) suggests these saws can increase the risk of occupational noise-induced hearing loss, according to National Institute for Occupational Safety and Health (NIOSH) recommendations.

**What’s Important About This Paper?**

Construction workers can be exposed to hazardous levels of noise while on the job. This paper characterizes the sound pressure levels of common power tools used to cut steel studs during the framing stage of commercial construction. Mean equivalent continuous sound pressure levels exceeded 100 dBA during cutting, and mean peak sound pressure levels exceeded 120 dBC, suggesting a need for noise exposure mitigation while cutting steel framing studs.

## Introduction

**Fig. 1.**
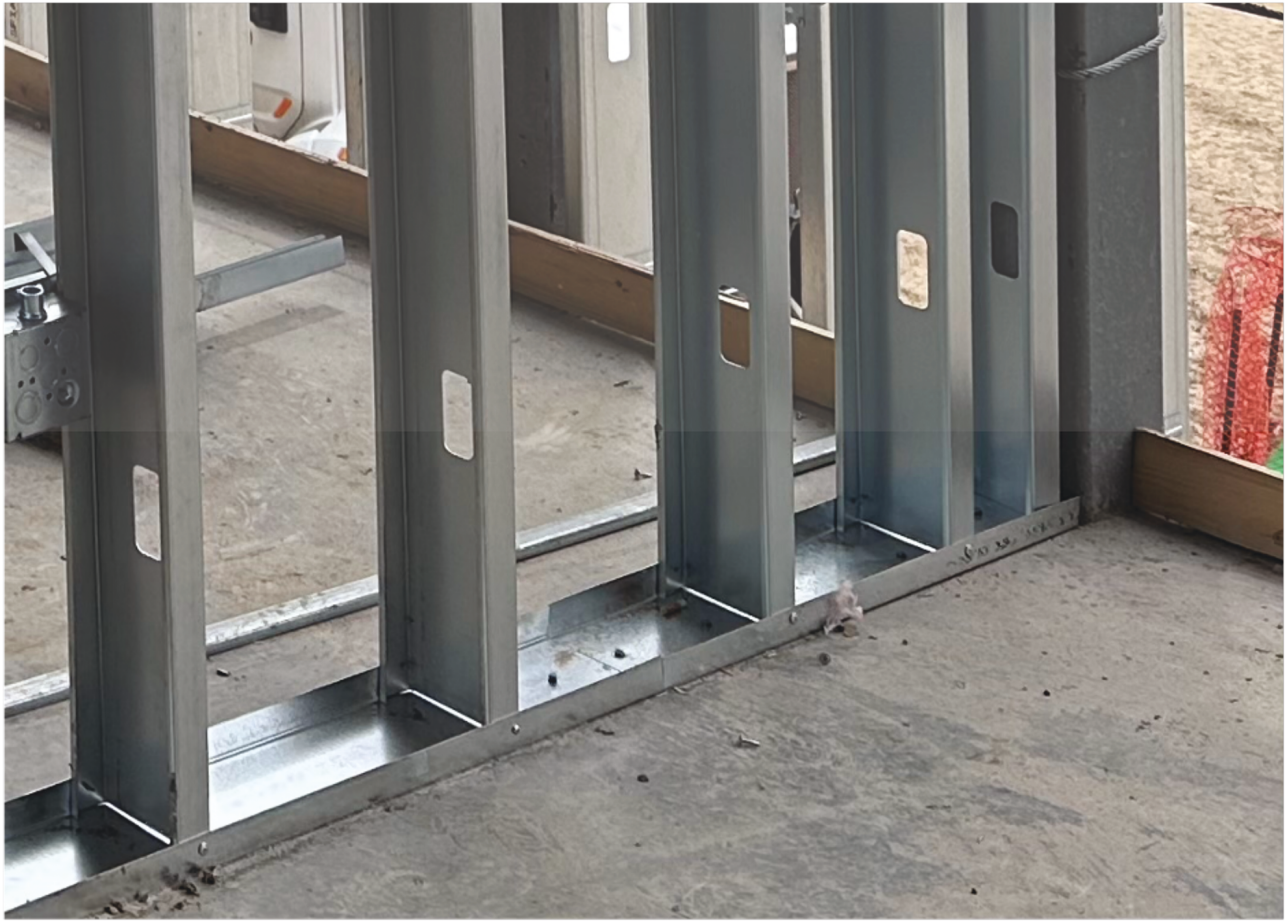
Example of steel stud framing. Vertical studs are attached to track mounted on the floor.

Commercial construction in the United States commonly uses steel studs as the primary framing components for interior and exterior building walls (Fig. 1). Steel studs used in commercial construction in the United States are typically produced with dimensional widths between 1.625 and 12.0 inches (41–305 mm) and dimensional depths between 1.0 and 2.5 inches (25–64 mm). Common thicknesses of the steel material range from 18 thousandths of an inch (mil) to 97 mil (0.454–2.454 mm). [All steel stud soft metric conversions are adapted from the Steel Stud Manufacturers Association (2002).] Steel studs typically arrive at a jobsite in various lengths that require the stud to be cut to a size suitable for installation. Light thickness studs such as 18–30 mil (0.454–0.753 mm) can be cut by hand with a pair of aviation snips; however, heavier thicknesses require the use of power saws for cutting. Almost exclusively, these power saws use a rotating circular blade or abrasive cut-off wheel to cut these studs quickly and efficiently.

While power saws used for cutting steel studs are anecdotally loud, little is known about their noise level production and worker exposure time on the jobsite, which are ultimately important factors in estimating the risk of occupational noise-induced hearing loss (NIHL) of the workers who use this equipment in their daily tasks. Occupational exposure limits for noise have been created by regulatory agencies and safety and health institutes to decrease risks of occupational NIHL. These exposure limits have been set based on a worker’s noise dose, which is a calculation of all hazardous noise exposures a worker receives during the course of their entire workday. Because each hazardous noise exposure during a workday cumulatively increases the worker’s daily noise dose, the characterization of sound levels from specific tools can help workers mitigate their overexposures and lower their daily noise dose to reduce the risk of occupational NIHL. This study investigated typical power saws used to cut steel framing studs at active construction sites to evaluate the sound pressure levels (SPLs) of these saws and exposure times at the worker’s hearing zone.

## Methods

All sampling was performed on volunteers at commercial construction sites. Institutional Review Board approval at Colorado State University was received prior to any recruitment. Volunteers with informed consent were recruited at commercial construction sites that were actively in the framing stage and were using steel studs as framing materials.

### Job site characteristics

Job sites where volunteers were recruited were commercial buildings under construction or renovation. All sites were selected from local framing contractors who gave pre-approval to allow noise sampling of their employees and who had framing jobs commencing in the near future. Sampling was conducted once a site became available where framing tasks had begun. Sites were a combination of new-build projects and renovation projects between 3,150 ft^2^ (300 m^2^) and 200,000 ft^2^ (18,000 m^2^) and were from 1 to 5 stories tall.

### Saw noise characterization

**Fig. 2.**
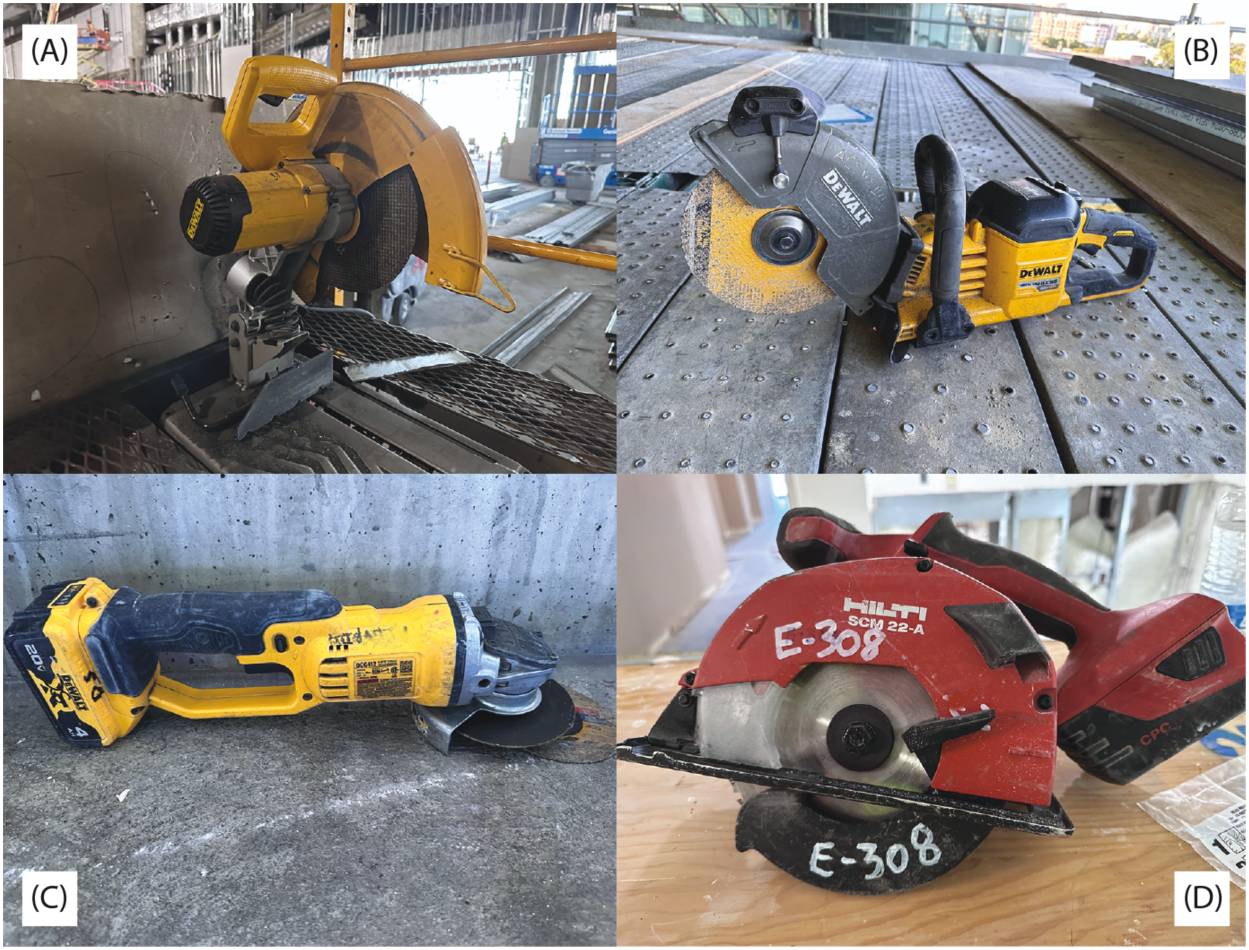
Examples of each saw type that was used at the study sites. A = Chopsaw; B = Cut-off Saw; C = Grinder; D = Cordless Circular Saw.

Sound pressure level data were collected for power saws typically used on the participants’ job sites. No modifications to a worker’s daily routine or tool selection were performed for the purpose of this study to ensure normal working conditions during sampling. Power saws were evaluated as generalized categories of tool type, not by manufacturer or age. Saws categorized as “chopsaw” were DeWalt D28710/15, as “cordless circular saw” were Hilti SCM 22-A, as “cut-off saw” were DeWalt DCS690, and as “grinder” were DeWalt DCG410. All chopsaws and cordless circular saws in the study were supplied by the employers, whereas all cut-off saws and grinders were supplied by individual employees. Specific saw models and paired blade/cut-off wheel configurations used by the workers in this study are shown in Supplementary Table S1. Examples of each saw are shown in Fig. 2.

Noise samples were obtained with one of two noise dosimeters (Larson-Davis Spark model 706RD and Larson-Davis Spartan model 730, Depew, NY, USA). The Spartan dosimeters were enabled with 12-second event sound recording for 6 of the 10 study days. All dosimeters were set to Occupational Safety and Health Administration Permissible Exposure Limit (OSHA PEL) criteria (90 dB criterion level, 90 dB threshold, 5-dB exchange rate, slow response) while recording. A-frequency weighting was used to calculate equivalent continuous sound pressure level (L_Aeq_), whereas C-frequency weighting was applied for peak sound pressure level (L_Cpeak_), which more accurately simulates the effects of high-amplitude and impulsive noise on the human ear (Meinke et al. 2022). All data were integrated and logged at 1-s resolution. Sound pressure level data were collected from the start of the work day until the end of the work day with lunch break data excluded. Calibration of each dosimeter was performed before and after each sampling day using Larson-Davis CAL150/200 calibrators (Depew, NY, USA) at 114 dB and 1000 Hz.

Dosimeters were attached to the participant’s shoulder with the microphone near the participant’s hearing zone (i.e. two-foot-wide sphere surrounding the head). As framers typically carry studs on their shoulder, the non-dominant shoulder was used to attach the dosimeter to avoid the worker carrying a stud on the dosimeter microphone. Noise data were downloaded via the dosimeter PC-based software (PCB Piezotronics G4 LD Utility for the Larson-Davis Spartan dosimeters, and PCB Piezotronics Blaze for the Larson-Davis Spark dosimeters, Depew, NY) and exported to spreadsheets for analysis.

Concurrent with SPL measurements, the investigators observed and documented times and descriptions of the tasks being performed by each participant. These observational data were then used to compare with the SPL data to identify a dB “signature” for saw cuts performed throughout the work day, i.e. the dB signatures were visually identified in the SPL time-stamped data.

### Cutting-time evaluations

Dosimeter clocks were synched with a smartphone clock used by the investigator. Times, saw type, and stud type were recorded in a field notebook by the investigator when a participant used a saw. Cutting times were observed and logged by the investigator for various types and sizes of steel studs used during study observation. Length of cutting time was measured with a smart phone stopwatch. The stopwatch was started when the worker pulled the saw trigger to start the saw, and the time stopped when the worker released the trigger at the end of each cut. The observed cutting times were subsequently correlated with the dB signatures identified in the SPL time-stamped data.

### Statistical analysis

All data from the dosimeters were exported into Microsoft Excel (ver. 16.76) spreadsheets. Statistical analyses were performed using R Statistical Software (v. 4.3.1; R Core Team 2023) with EnvStats package (Millard 2013).

Means of all noise metrics, *L*_*x*_, were calculated as log-transformed means as shown in Eq. 1, as described in International Organization for Standardization (ISO) 9612:2009.


$$\begin{array}{l} L_{x\left(avg\right)}=10log_{10}\left(\frac{1}{N}\underset{i=1}{\overset{N}{\sum }}\;10^{0.1L_{xi}}\right) \end{array}$$ (1)

where:

*L*_*x(avg)*_ is the log-transformed mean of all L_Aeq_ or L_Cpeak_ data for a specific saw

*L*_*xi*_ is the individual 1-s measured L_Aeq_ or L_Cpeak_ for the event

*i* is the 1-s sample number

*N* is the total number of 1-s measurements

## Results

For these saw noise characterizations, a total of 23 construction workers were sampled at 6 sites over the course of 10 d. Across all study sites, 4 different types of power saws (chopsaw, cordless circular saw, cut-off saw, and grinder) were used by the workers to cut steel studs. In all, a total of 9,527 1-s noise samples of power saw use were recorded by the participant dosimeters. No overloading of the microphones from saw noise was detected by the dosimeters.

**Fig. 3.**
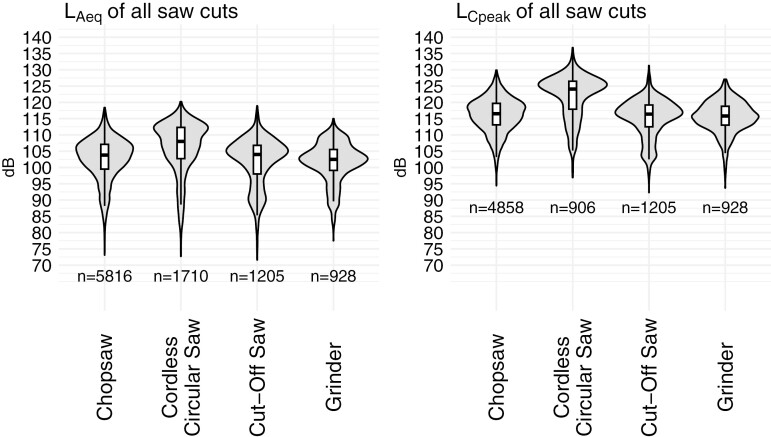
Violin plots of noise metrics for each power saw found on each jobsite for the study. Data presented as: L_Aeq_ (A-weighting L_eq_) and L_Cpeak_ (C-weighting L_peak_). Boxplots: Horizontal line = median, boxes = interquartile range (IQR), whiskers = 1.5*IQR. Each sample (n) = 1 second.

Violin plots with medians of all L_Aeq_ and L_Cpeak_ data for each saw are shown in Fig. 3. Summary statistics for each saw category are reported in Table 1. Of particular note, the central tendencies for all metrics of the cordless circular saw are conspicuously higher than all other saws.

**Table 1.** Noise metric summary statistics for each saw category used in the study.

Saw	Mean L_Aeq_ (dB)	Mean L_Cpeak_ (dB)	Median L_Aeq_ (dB)	Median L_Cpeak_ (dB)	SD L_Aeq_ (dB)	SD L_Cpeak_ (dB)
Chopsaw	106.3	118.9	103.8	116.5	6.6	5.3
Cordless Circular Saw	110.4	125.2	108.0	124.1	8.0	6.7
Cut-off Saw	105.9	118.3	104.0	116.4	7.4	5.8
Grinder	104.7	117.8	102.5	115.8	6.1	4.9
All Saw Data Combined	107.2	120.1	104.2	116.9	7.1	5.8

Means are log-transformed dB data. L_Aeq_ = A-weighting L_eq_ and L_Cpeak_ = C-weighting L_peak_.

Average times to cut steel studs varied by saw type and stud dimension. The overall mean time to cut a stud for any saw was 13.2 s. The mean length of stud cutting time per worker per day of any saw use was 371.5 s (range 8–1105 s; SD 323.4; median 380). The length of time to cut specific stud sizes by saw type is reported in Supplementary Table S2 for those that were evaluated during the study. Due to the large number of stud/saw combinations and the inability to directly monitor every stud cut, as well as numerous other variables (e.g. cutting multiple studs at once), not all stud/saw combinations are reported.

Based on all data collected for this study, the chopsaw comprised 56.9% of total saw usage, the cordless circular saw comprised 26.2%, the cut-off saw comprised 11.1%, and the grinder comprised 5.8% of total saw usage.

## Discussion

Construction workers are well-documented to be exposed to excessive levels of noise in their work (Neitzel et al. 1999; Kerr et al. 2002; Suter 2002; Lewkowski et al. 2018). The data presented in this study demonstrate that power saws used for steel stud framing may contribute to excessive noise in commercial construction framers and may be hazardous at the exposure durations seen in this study.

The noise data shown in Fig. 3 represent all recorded data of saw operation, from the time the saw operator pulled the saw trigger and initiated the cut, to the end of the cut when the saw trigger was released. This data recording included not only the noise level produced during the cut (~105–130 dBA) but also the noise level of the saw prior to or following contact with the stud when the blade/cut-off wheel was rotating but not touching the stud (~90–95 dBA). The saw motors produced a continuous noise while running, but the contact of the blade/cut-off wheel with the stud created a non-continuous noise that could be described as complex due to the stochastic vibrational contact of the saw blade/cut-off wheel against the sides of the kerf as the metal was being cut (Tönshoff et al. 1981).

Of the 4 types of power saws observed for this investigation, 3 had similar noise levels while cutting a generalized steel stud, and a fourth saw, the cordless circular saw, had conspicuously higher levels for all noise metrics on the logarithmic dB scale (Fig. 3, Table 1). Note that because each 1-s sample in the data set is correlated to the samples immediately before and after each recording, and therefore, the data are not independent of each other, statistical comparisons through ANOVA testing are not possible. Central tendencies for each of the saws are used to add support to the visual data plots that the cordless circular saw had higher overall noise metrics than the other 3 saw types (Table 1); however, no formal statistical conclusions can be drawn from these comparisons.

The L_Aeq_ data provide information and guidance that relates most closely to regulatory compliance and protective recommendations; however, the calculations that provide L_Aeq_ data do not adequately integrate high L_Cpeak_ exposures. As such, instantaneous peaks in noise energy that are captured by the peak detector in the dosimeter occur so briefly (in milliseconds) that they have little effect on an L_eq_ integration calculation, even with very high peaks such as 130 dB as produced by the saws in this investigation (Qiu et al. 2020; Zhang et al. 2022). Although L_peak_ data do not affect regulatory compliance where agencies such as OSHA have a ceiling limit of 140 dB that is merely recommended, researchers have nonetheless reported that high peak exposures, such as those demonstrated with the power saws in this study, can negatively impact hearing, even when a worker’s L_Aeq_ exposures fall within compliance (Kelsall 2006; Goley et al. 2011).

Mean L_Aeq_ in this study for all saws was 107.2 dBA. NIOSH recommendations consider a daily exposure time of >179 s at 107 dBA to be hazardous to workers’ hearing (NIOSH 1998). At 104 dBA, a level less than the mean L_Aeq_ of the quietest saw in the study (Table 1), NIOSH daily recommended exposure limits would be exceeded with >357 s of use (NIOSH 1998). The workers in this study used saws on average 371.5 s per day, putting more than half of these workers at hearing loss risk, a number which is likely understated since the quietest tool (the grinder) was used the least in this study (5.8%).

**Fig. 4.**
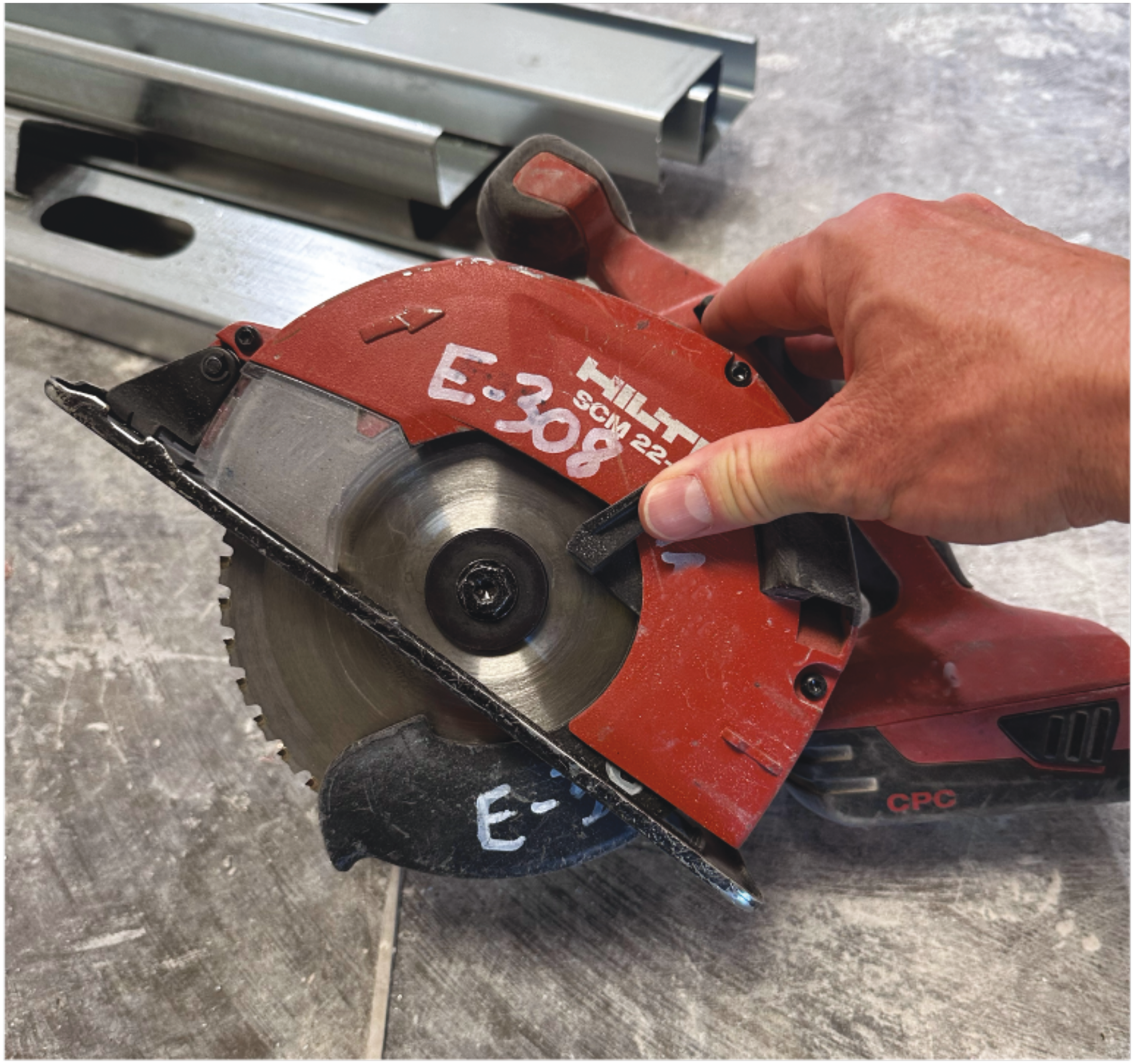
Detail of the metal-cutting blade on the cordless circular saw.

While this characterization is not intended to compare or contrast the hazards of using each specific type of saw, Fig. 3 visually indicates that the cordless circular saw had higher noise levels than all other saws. Of note with this saw, it was the only saw to use a metal-cutting blade as opposed to an abrasive cut-off wheel used in the other saws (Fig. 4). Because no sites in this study used the cordless circular saw with an abrasive cut-off wheel, it is unknown whether the higher noise levels of this saw are due to the blade, or to the saw itself. Interestingly, this saw also had generally faster cutting times than other saw types (Supplementary Table S2). It is unknown if the blade used with this saw contributed to the cutting speed, or if it was a factor of other engineering or operator variables, such as saw motor power or operator cutting aggressiveness.

Stud cutting times were measured for all studs found on the study sites to calculate noise exposures from individual cuts (Supplementary Table S2). Due to the high variability in cutting times, comparing efficiency among saws for different stud types would require additional investigations. The mean time to cut any stud size with any saw in this study was 13.2 s, but ranged widely depending on the size of the stud and the type of saw used (2.5–106.0 s; Supplementary Table S2). This wide variation makes it difficult to estimate the total number of studs a worker could cut each day before exceeding occupational exposure limits. With an average saw use of 371.5 s in this investigation, it may not take many stud cuts for a worker to be overexposed at ~107 dB while cutting studs without properly worn hearing protectors or adequate engineering controls.

Faster saws may decrease the worker’s overall noise exposure for the day, however, even if that saw is louder. As shown in Supplementary Table S2, the cordless circular saw, while substantially louder than the other saws, had faster mean cutting times for the same stud size than other saws. This presents a question on whether a faster saw can decrease the overall noise exposure by subjecting the worker to more intense—but shorter-duration—sound energy. Due to the logarithmic nature of dB values and the equal energy hypothesis, every 3 dB increase presents a doubling of noise energy, and therefore, a doubling of noise exposure (NIOSH 1998). Thus, if a saw cuts a stud exactly twice as fast, but is more than 3 dB louder, then the shorter cut could be more damaging to hearing due to higher sound intensity. In this study, the cordless circular saw had mean L_Aeq_ values >4 dB louder than all other saws (Table 1), so it would need to cut a stud >2 times faster to offset the higher sound intensity. Based on stud cutting times presented in Supplementary Table S2, the cordless circular saw cutting at >2 times faster than other saws does not appear to be a trend, except in comparison with the grinder. Thus, in the instance of the cordless circular saw used in this study, its increased speed at cutting does not generally decrease noise exposure, and conversely likely increases overall noise exposure in the user. Alternatively, the cut-off saw cuts studs faster than other saws in some instances with similar mean sound intensity (Table 1 and Supplementary Table S2). Thus, this saw has the potential to not only cut faster but can decrease noise exposure in the saw operator when compared to using other saws. This presents a dilemma, however, in that increased production may lead to additional studs cut throughout the day, thereby adding to unintended additional noise exposure.

The circular saw with metal cutting blade (Fig. 4), despite its high noise levels, presents an interesting case in that this blade type did not produce sparks while cutting steel studs in this investigation. This decrease in fire hazard, therefore, meant that a hot work permit was not required for use with this saw, where a permit may otherwise be mandated that specifies training for the operator and fire suppression equipment nearby (i.e. a fire extinguisher) to prevent a fire from the sparks (NFPA 2024). In some instances, hot work permit requirements may not be able to be fulfilled, leaving the workers to rely on the louder equipment that does not produce sparks while cutting studs.

To mitigate the exposure to the hazardous noise produced by these saws, workers can use hearing protectors, an acceptable but least preferred method to control a hazard (Morris and Cannady 2019; ANSI/ASSP 2020; Meinke et al. 2022). Hearing protectors of various brands and styles were available to the workers at all study sites; however, some workers chose not to use any hearing protectors while using the saws, and most who did use them were observed not wearing them fully inserted. The nature of commercial framing work can make full-time use of hearing protectors challenging (Suter 2002), where workers commonly must communicate with each other over long distances, such as yelling from a scissor lift near a 30-ft (10-m) ceiling down to the saw operator at the floor level. To spend the time to insert foam ear plugs intermittently, which may take 20 s to insert properly, for a saw cut that takes only ~13 s to complete, may be seen as an inconvenience by the worker. With a potential lack of education on the irreversible effects of noise-induce hearing loss, workers are oftentimes making the wrong decision and choosing speed or convenience at the expense of hearing. As such, quieter power saws would mitigate the hazard and allow workers to focus more on their work and less on hearing protection.

## Conclusion

The results of this study demonstrate that power saws may vary in their noise level production and cutting efficiency while cutting steel studs on commercial construction sites. A common theme among all saws used in commercial framing is the high noise levels of the saws while cutting steel studs. Although the cordless circular saw had higher average noise levels than all other saws in this study, these data demonstrate that all saws observed in this study produced hazardous noise levels for the saw operators based on their average exposure duration and may contribute to occupational noise-induced hearing loss in this group of workers (Neitzel et al. 1999; Suter 2002; Leensen and Dreschler 2015). These results suggest that engineering controls for this equipment should be developed to help protect the hearing of commercial construction framers.

## Supplementary material

Supplementary material is available at *Annals of Work Exposures and Health* online.

wxae054_suppl_Supplementary_Tables

## Data Availability

De-identified data that support the findings of this study are available by reasonable request to the corresponding author, DS.
