# Supplementary material for: Power saw noise levels during steel stud cutting tasks on commercial construction sites: a tool characterization from a worker exposure standpoint
Source: Ann Work Expo Health. 2024 Jun 24;68(8):874–80. doi: 10.1093/annweh/wxae054 (PMC11427544; doi:10.1093/annweh/wxae054)
Supplement: wxae054_suppl_Supplementary_Tables [file wxae054_suppl_supplementary_tables.pdf]

Power saw noise levels during steel stud cutting tasks on commercial construction sites: A tool characterization from a worker exposure standpoint

David Schutt[1], Tiffany Lipsey[2], Mike Van Dyke[3], William J. Brazile[1]

[1]*Department of Environmental and Radiological Health Sciences, Colorado State University, 1681 Campus Delivery, Fort Collins, CO, 80523-1681, United States*

[2] *Department of Health and Exercise Science, Colorado State University, 1582 Campus Delivery, Fort Collins, CO, 80523-1582, United States*

[3]*Department of Environmental and Occupational Health, Colorado School of Public Health, CU Anschutz Medical Campus, Aurora, CO, 80045, United States*

Corresponding Author: David Schutt, David.Schutt@colostate.edu

Supplemental Material

Table S1. Saw and blade/cut-off wheel configurations for the power saws used to cut steel studs at the study sites. *n* = sample size.

**Saw Type**	***n***	**Saw Manufacturer**	**Saw Model**	**Blade/Cut-off Wheel Type(s)**
Chopsaw	5816	DeWalt	D28710/15	Hilti 436732 Drywall Stud Cutting Wheel<br>DeWalt DWA8001 Metal Cutting Wheel
Cordless Circular Saw	1710	Hilti	SCM 22-A	Hilti SCBM MU 6.5” 40t Blade
Cut-off Saw	1205	DeWalt	DCS690	DeWalt DWAFV8918 Cutoff Wheel
Grinder	928	DeWalt	DCG412	DeWalt DW8062 Cutoff Wheel

Table S2. Cutting times (sec) to cut steel studs based on the web size and thickness of each stud, per saw type observed. Dimension = stud web measurement; *n* = sample size of the specific stud type/saw combination; SD = standard deviation; (-) = no event observed.

			**Seconds to Cut One Stud at a Time**				**Seconds to Cut Two Studs at a Time**			
**Tool**	**Dimension**	**Thickness**	***n***	**Mean**	**SD**	**Range**	***n***	**Mean**	**SD**	**Range**
Grinder	3.625 inch (92 mm)	54 mil (1.366 mm)	14	23.2	5.4	11.6-30.7	-	-	-	-
Chopsaw	3.625 inch (92 mm)	18 mil (0.454 mm)	3	4.5	1.9	2.5-6.4	5	5.8	2.7	3.4-10.2
Chopsaw	3.625 inch (92 mm)	33 mil (0.835 mm)	3	2.5	0.8	2.0-3.4	-	-	-	-
Chopsaw	6 inch (152 mm)	33 mil (0.835 mm)	13	9.7	3.5	4.5-18.0	-	-	-	-
Chopsaw	6 inch (152 mm)	43 mil (1.088 mm)	32	8.4	3.4	3.7-15.3	18	18.4	4.4	12.5-32.0
Chopsaw	6 inch (152 mm)	54 mil (1.366 mm)	14	28.9	11.3	11.0-45.0	1	82.2	0	82.2-82.2
Chopsaw	6 inch (152 mm)	97 mil (2.454 mm)	6	57.7	26.1	34.4-106.0	-	-	-	-
Chopsaw	8 inch (203 mm)	33 mil (0.835 mm)	12	11.0	4.3	6.5-22.1	6	15.9	2.8	13.4-21.0
Cordless Circular saw	3.625 inch (92 mm)	30 mil (0.753 mm)	1	6.1	0	6.1-6.1	8	10.0	2.8	6.8-14.0
Cordless Circular saw	3.625 inch (92 mm)	43 mil (1.088 mm)	-	-	-	-	8	10.3	2.2	6.5-13.0
Cordless Circular saw	6 inch (152 mm)	30 mil (0.753 mm)	5	9.8	1.3	8.0-11.2	6	15.0	2.9	10.8-19.4
Cordless Circular saw	6 inch (152 mm)	33 mil (0.835 mm)	2	7.5	6.3	3.1-12.0	-	-	-	-
Cordless Circular saw	6 inch (152 mm)	43 mil (1.088 mm)	25	6.7	3.2	2.8-17.3	-	-	-	-
Cordless Circular saw	8 inch (203 mm)	33 mil (0.835 mm)	1	5.0	0	5.0-5.0	-	-	-	-
Cordless Circular saw	8 inch (203 mm)	43 mil (1.088 mm)	3	8.3	2.1	6.0-10.0	-	-	-	-
Cut-Off Saw	3.625 inch (92 mm)	54 mil (1.366 mm)	3	4.7	2.9	3.0-8.0	-	-	-	-
Cut-Off Saw	6 inch (152 mm)	54 mil (1.366 mm)	6	8.7	1.1	7.6-10.6	-	-	-	-
Cut-Off Saw	10 inch (254 mm)	54 mil (1.366 mm)	8	15.7	5.2	9.7-27.3	5	29.5	3.2	25.8-34.0
